# Compassion fatigue among nurses working on an adult emergency and urgent care unit ^*^

**DOI:** 10.1590/1518-8345.2973.3175

**Published:** 2019-10-07

**Authors:** Elisabete Maria das Neves Borges, Carla Isabel Nunes da Silva Fonseca, Patrícia Campos Pavan Baptista, Cristina Maria Leite Queirós, María Baldonedo-Mosteiro, María Pilar Mosteiro-Diaz

**Affiliations:** 1 Escola Superior de Enfermagem do Porto , Porto , Portugal .; 2 Centro Hospitalar de São João , Porto , Portugal .; 3 Universidade de São Paulo , Escola de Enfermagem , Departamento de Orientação Profissional , São Paulo , SP , Brasil .; 4 Universidade do Porto , Faculdade de Psicologia e de Ciências da Educação , Porto , Portugal .; 5 Universidad de Oviedo , Facultad de Psicología , Oviedo , Espanha .; 6 Universidad de Oviedo , Departamento de Medicina,Enfermería , Oviedo , Espanha .

**Keywords:** Compassion Fatigue, Nurses, Hospitals, Emergency Service, Professional Exhaustion, Work, Fadiga por Compaixão, Enfermeiros, Hospitais, Emergências, Esgotamento Profissional, Trabalho, Desgaste por Empatía, Enfermeros, Hospitales, Urgencias Médicas, Agotamiento Profesional, Trabajo

## Abstract

**Objective:**

to assess compassion fatigue levels among nurses and its variation according socio-demographic and professional characteristics.

**Method:**

quantitative, descriptive and cross-sectional study, with 87 nurses from an emergency and urgent care unit for adults from a university hospital. A socio-demographic and professional questionnaire, along with the Professional Quality of Life Scale 5 were used. Data analysis was performed using descriptive and inferential statistics.

**Results:**

compassion satisfaction presents the highest means, followed by burnout and secondary traumatic stress. Among the participants, 51% presented a high level of compassion satisfaction, 54% a high level of burnout, and 59% a high level of secondary traumatic stress. Older participants presented higher score of compassion satisfaction, and younger nurses, women, nurses having less job experience and nurses without leisure activities showed higher means of secondary traumatic stress.

**Conclusion:**

we found compassion fatigue, expressed in the large percentage of nurses with high levels of burnout and secondary traumatic stress. Fatigue is related to individual factors such as age, gender, job experience and leisure activities. Doing research and understanding this phenomenon allow the development of health promotion strategies at work.

## Abstract (pt)

**Objetivo:**

avaliar o nível de fadiga por compaixão em enfermeiros e sua associação em função de características sociodemográficas/profissionais.

**Método:**

estudo quantitativo, descritivo e transversal, com 87 enfermeiros de um serviço de urgência e emergência de adultos, de um hospital universitário. Aplicaram-se um questionário sociodemográfico/profissional e a escala *Professional Quality of Life Scale 5* . Para a análise dos dados, recorreu-se à estatística descritiva e inferencial.

**Resultados:**

verificou-se que a satisfação por compaixão apresenta as médias mais elevadas, seguida do *burnout* e do estresse traumático secundário. Encontraram-se no nível elevado 51% dos enfermeiros na satisfação por compaixão, 54% no *burnout* e 59% no estresse traumático secundário. Os participantes com mais idade apresentaram médias superiores de satisfação por compaixão, enquanto os do sexo feminino, mais novos, com menos tempo de experiência profissional e que não tinham atividades de lazer evidenciaram média superior de estresse traumático secundário.

**Conclusão:**

existe fadiga por compaixão expressa na grande percentagem de enfermeiros com elevados níveis de *burnout* e de estresse traumático secundário. A fadiga depende de fatores individuais como idade, sexo, experiência profissional e atividades de lazer. A pesquisa e a compreensão desse fenômeno permitem o desenvolvimento de estratégias de promoção de saúde no trabalho.

## Abstract (es)

**Objetivo:**

evaluar el nivel de fatiga por compasión de los enfermeros y su asociación con las características sociodemográficas/profesionales.

**Método:**

se trata de un estudio cuantitativo, descriptivo y transversal, realizado entre 87 enfermeros de un servicio de urgencias y emergencias de adultos de un hospital universitario. Se aplicó un cuestionario sociodemográfico/profesional y la Escala de Calidad de Vida Profesional (ProQOL5). Para el análisis de los datos se utilizaron las estadísticas descriptiva e inferencial.

**Resultados:**

se comprobó que la satisfacción por compasión alcanza medias elevadas, seguida de burnout y de estrés traumático secundario. En el 51% de los enfermeros se halló satisfacción por compasión, en el 54%, burnout y en el 59%, estrés traumático secundario. Los participantes con más edad presentaban medias superiores de satisfacción por compasión y los del sexo femenino, más jóvenes, con menos tiempo de experiencia profesional y que no participaban de actividades de ocio, evidenciaban una media superior de estrés traumático secundario.

**Conclusión:**

existe fatiga por compasión expresa en gran porcentaje de los enfermeros con niveles elevados de burnout y de estrés traumático secundario. La fatiga depende de factores individuales como edad, sexo, experiencia profesional y actividades de ocio. La investigación y la comprensión de este fenómeno permiten el desarrollo de estrategias para la promoción de la salud laboral.

## Introduction

Health, safety, and well-being of health professionals are the focus of worldwide attention, due emotional demands of their task and the importance they have on the productivity, competitiveness and sustainability of organizations ^1 - 3^ .

Compassion fatigue, which is considered one of the greatest threats to the mental health of health professionals ^4 - 7^ , is defined as “the natural, consequent behaviors and emotions resulting from knowing about a traumatizing event experienced by a significant other – the stress resulting from helping or wanting to help a traumatized or suffering person” ^8^ . Lately, the Professional Quality of Life model describes compassion fatigue as the combination of high burnout, secondary traumatic stress and low compassion satisfaction ^9^ .

Different factors contribute to compassion fatigue, with emphasis on personality, education, job experience, personal quality of life and, at the organizational level, the specificity of the tasks and the changes of the health system ^6 , 10^ . Due to the considerable demand and frequent contact with traumatic situations, nursing work in emergency and urgent care makes nurses susceptible to feel the pain of their patients, increasing compassion fatigue ^11 - 13^ .

The expressions of compassion fatigue are varied and have not always been valorized. They develop over time and compromise not only the physical, psychological, cognitive and spiritual health of professionals, but also their personal, social and professional life, with a negative impact on their well-being and quality of life, as well as on the health institutions and on the quality of care provided ^4 - 5 , 14 - 16^ . Considering that nurses have emotionally demanding tasks and work under stressful conditions ^17 - 20^ , this study aimed to assess compassion fatigue levels among nurses and its variation according socio-demographic and professional characteristics.

## Method

This is a quantitative, descriptive and cross-sectional study conducted from May to July 2017, with Portuguese nurses from an emergency and urgent care unit from a university hospital in the city of Porto, Portugal. The inclusion criterion was being a nurse with job experience more than 6 months. A convenience sample from a population of 93 nurses was selected, participating 87 nurses, which represents a 94% adhesion rate.

The data was collected through a self-reporting instrument containing socio-demographic questions (gender, age, marital status, children, academic qualification and leisure activities), job questions (years of job experience, work contract, working schedule, presence of dependents, monthly income, and a question asking if they considered their work stressful), and the Professional Quality of Life Scale (ProQOL5), translated and adapted for the Portuguese population ^9 , 21^ . This instrument evaluates compassion fatigue and consists of 30 items organized on 3 subscales, each one composed of 10 items that evaluate three distinct phenomena: compassion satisfaction, burnout and secondary traumatic stress. Each item has a statement where the participant assigns a score on a Likert scale that ranges from 1 (Never) to 5 (Very often). Compassion fatigue results from high burnout and high secondary traumatic stress. This scale was chosen because it is currently one of the most used to evaluate compassion fatigue, being interesting for researchers since it includes the positive component – compassion satisfaction – and not only the negative components ^9^ .

This study was approved by the Research Ethics Committee for Health, by the Board of Directors of the university hospital and by the authors of the Portuguese version of the Professional Quality of Life Scale - ProQOL5. The study is part of the project “ *INT-SO – From work contexts to occupational health of nursing professionals, a comparative study between Portugal, Brazil and Spain* ”, of the NursID: Innovation & Development in Nursing: Center for Health Technology and Services Research (CINTESIS).

After an informal contact with the chief nurse of the emergency and urgent care unit where the research was conducted, the application of the instruments was scheduled. One of the researchers established direct contact with the potential participants and presented the study information document, the informed consent term and the instrument of data collection. The nurses who agreed to participate in the study returned the instruments to the same researcher in a closed envelope, in order to guarantee their anonymity.

The data were analyzed through descriptive and inferential statistics in the Statistical Package for the Social Sciences, SPSS version 24. The analysis was performed using absolute and relative frequencies, measures of central tendency such as mean, median, maximum, minimum and Standard Deviation, the Pearson’s correlation coefficient, the parametric Student’s t-test for independent samples, and the Mann-Whitney non-parametric test. Statistical analysis set the significance level at p <0.05 (95% confidence level). Reliability of the subscales was evaluated using the Chronbach’s alpha coefficient and normality was evaluated using the Kolmogorov-Smirnov test.

To calculate the cut-off points of the Professional Quality of Life Scale - ProQOL5, the guidelines of the author ^9^ were followed. The raw scores of the subscales compassion satisfaction, burnout and secondary traumatic stress were converted in Z scores and these in t-scores. The forced conversion of raw scores to obtain M=50 and SD=10 makes it possible to compare the score of the three dimensions and to compare the results with other studies.

## Results

Regarding the socio-demographic and job characteristics, 57 (65.5%) of the nurses were female; the mean age was 37.1 years (SD=6.3), with a median of 36, a minimum of 25 and maximum of 52. Fifty (57.5%) nurses had no partner, 42 (48.2%) had children, 80 (94.1%) had a teaching license degree and 5 (5.6%) had a graduate degree, 84 (96.6%) had a permanent contract, 85 (97.7%) worked rotating shifts. The mean job experience was 13.9 (SD=6.1) years, with a minimum of 3, a maximum of 31 and median of 11 years; 55.2% of the participants had no dependents and 12.6% depended only on their monthly income. Regarding job stress, 86 (96.6%) of the participants considered their work stressful, but 56 (64.4%) had some kind of leisure activity outside working hours; the most cited activity was physical exercise.

The Cronbach’s alpha coefficients of the Professional Quality of Life Scale - ProQOL5 in the subscales compassion satisfaction (0.90), burnout (0.77) and secondary traumatic stress (0.82) were similar to those obtained in the original ^9^ and in the Portuguese version ^21^ , which had score of 0.88; 0.75; 0.81 and 0.86; 0.71; 0.83, respectively.

The analysis of the dimensions of professional quality of life reveals that compassion satisfaction has the highest mean score, followed by burnout. Secondary traumatic stress has the lowest score ( Table 1 ). Correlation analysis between dimensions revealed that the correlation between compassion satisfaction and burnout is negative and strong, between compassionate satisfaction and secondary traumatic stress it is negative but weak, and between burnout and secondary traumatic stress is positive but weak ( Table 1 ).

**Table 1:** Descriptive and correlational analysis of nurses’ compassion fatigue dimensions, Porto, Portugal, 2017

Dimensions of the ProQOL5* (10-50)	Min	Max	x̄	SD	Compassion Satisfaction	Burnout
Compassion satisfaction	22	48	37.1	5.9		
Burnout	16	38	26.0	5.6	-0.602 (0.000)*	
Secondary traumatic stress	11	39	23.9	5.5	-0.116	0.456 (0.000) ^†^

*ProQOL5 = Professional Quality of Life Scale; ^†^ value obtained through the Pearson’s correlation coefficient

Based on the cut-off points, it was verified ( Table 2 ) that 51% of the nurses have a high level of compassion satisfaction and 20% have a low level. For burnout, 54% present a high level and 24% a low level and in secondary traumatic stress, 59% present a high level and 20% a low level. If we create a group joining medium and high levels of the different subscales, we find a value of 81% in compassion satisfaction, 76% in burnout and 80% in secondary traumatic stress.

**Table 2:** – Cut-off points of the Professional Quality of Life Scale – ProQOL5 and frequencies of the scores in the subscales compassion satisfaction, burnout and secondary traumatic stress, of nurses, Porto, Portugal, 2017

Dimensions of the Professional Quality of Life Scale – ProQOL5*	Percentile cut off (tscores)			Level– N (%)		

	25	50	75	Low	Medium	High
Compassion satisfaction	42.9	51.4	56.4	17 (19.5)	26 (29.9)	44 (50.6)
Burnout	42.8	48.1	58.8	21 (24.1)	19 (21.8)	47 (54.0)
Secondary traumatic stress	43.0	48.3	59.1	17 (19.5)	19 (21.8)	51 (58.6)

*ProQOL5 = Professional Quality of Life Scale

Regarding the socio-demographic and job characteristics, it was verified that among the 51% with a high level of compassion satisfaction, the majority are women (53%), aged 36 or over (59%), without a partner (56% ), graduated (50%), with job experience less than 11 years (53%), working less than 9 years (54%) in the current unit, and considering their work as stressful (51%).

Among the nurses, 54% presented high burnout. Of these, most are women (54%), under the age of 35 (61%), without a partner (58%), with a graduate/Masters/Doctorate degree, with 12 years or more of job experience (55%), working in the current unit of 10 years or more (64%) and most considering their work as stressful (55%).

Among the 59% with a high level of secondary traumatic stress, the majority are women (67%), under the age of 35 (74%), without a partner (64%), graduated (61%), with less than 11 years of job experience (68%), working in the current unit less than 9 years (69%) and most considering work as stressful (60%).

The comparative analysis according to socio-demographic and job characteristics revealed statistically significant differences related with age, gender, job experience and leisure activities ( Table 3 ). Regarding the age, nurses aged 36 years or older presented higher means of compassion satisfaction and lower burnout. Younger nurses, women, and with 11 years or less of job experience showed higher score of secondary traumatic stress. The nurses who did not engage in leisure activities presented higher score of burnout and secondary traumatic stress.

**Table 3:** – Comparative analysis of the ProQOL5 * according nurses’ age, gender and leisure activities, Porto, Portugal, 2017

ProQOL5*	Variable	N	x̄ (SD)	p ^†^
Compassion satisfaction	≤ 35 years	38	46.7 (10.7)	-0.006
	≥ 36 years	49	52.5 (8.6)	

Burnout	Leisure Activity -Yes	56	48.3 (9.2)	0.041
	Leisure Activity -No	31	52.9 (10.7)	

Secondary traumatic stress	≤ 35 years	38	53.1 (9.8)	0.008
	≥ 36 years	49	47.5 (9.4)	
	Female	57	51.9 (9.1)	0.011
	Male	30	46.2 (10.6)	
	≤ 11 years	38	52.6 (10.6)	0.031
	≥ 12 years	49	47.9 (9.0)	
	Leisure Activity -Yes	56	48.3 (9.4)	0.041
	Leisure Activity -No	31	52.9 (10.4)	

*ProQOL5 = Professional Quality of Life Scale; ^†^ value obtained through the Student’s t-test

No differences were found according marital status, children, academic qualification, job experience in the current unit, presence of dependents, family income and perceiving work as stressful.

## Discussion

The mean score found through the raw scores of the subscales compassion satisfaction, burnout, and secondary traumatic stress are similar to those of other investigations ^22 - 23^ . The same was found for the score of compassion satisfaction, burnout, and secondary traumatic stress regarding cut-off points ^4 , 9 , 21^ . In general, the results of this study demonstrate, as did the aforementioned studies, the predisposition of nurses to develop high levels of compassion fatigue. Several studies have shown the emotional costs of caring for people in distress, emphasizing the association between compassion fatigue and job stress, especially when stress is chronic and becomes burnout ^18 , 24 - 26^ , as well as when situations are emotionally draining and lead to primary post-traumatic stress ^10 , 14 - 15 , 20 , 27^ . In fact, the possibility of nurses being affected by their experiences, associated with the altruistic character and the empathic concern that characterizes the professional relationship established with the patients, represent risk factors for the development of compassion fatigue and, consequently, are a threat to the mental health and well-being of these professionals ^6 - 7^ .

Regarding the variation of compassion fatigue according to socio-demographic and job characteristics, these data corroborate studies suggesting that women present higher score of secondary traumatic stress than men. This might be related to the empathic ability of women to connect with their patients and feel their fears and traumas ^9^ . However, the same does not occur with age, that in other studies did not reveal a significant difference ^9 , 21 - 22^ , whereas in this study nurses aged 36 years and older presented higher score of compassion satisfaction, but lower score of secondary traumatic stress. Moreover, younger professionals presented lower score of compassion satisfaction and higher score of secondary traumatic stress, results similar to those of other researches ^28^ . Maybe this is due to a strong ability to adapt to situations, as well as the healthy worker effect, that is, nurses who are effectively suffering from psychological distress do not volunteer to participate in studies or may have abandoned their profession.

It was also verified that older nurses, especially women, present higher levels of compassion satisfaction, which corroborates the results of other studies ^22^ and suggests that women have a higher prevalence of compassion satisfaction and greater ability to take care of those who suffer. Nurses with job experience less than 11 years presented higher score of secondary traumatic stress, which is probably because they are less experienced and identify themselves with patients more easily. This leads to the belief that compassion fatigue decreases with years of job experience ^7 , 29^ , and that it may be related to an adaptive ability that is not yet so noticeable in less experienced nurses. Nurses who do not have leisure activities are more exposed to burnout and secondary traumatic stress, confirming the idea that professionals who do not invest in their personal quality of life are at greater risk to develop compassion fatigue ^6^ , because they focus their whole life on work, and when it does not meet expectations they are more vulnerable to burnout and psychological distress.

Despite of the limitations of the cross-sectional design and the convenience sample, which do not allow extrapolation of results to other contexts, this research can contribute to the study of compassion fatigue as a phenomenon that has been increasingly considered as a threat to the nurses’ mental health ^5 , 7^ . Thus, it is possible to alert nurses and hospital managers about the importance of monitoring the mental health of health professionals, and try to ensure that their emotional and psychological state is not much affected by the care they provide to patients, so that they can continue to provide an optimal level of care.

## Conclusion

The study revealed the presence of medium and high levels of compassion satisfaction, burnout and secondary traumatic stress in the sample studied. In addition, the results show that compassion fatigue is related to personal factors such as age, gender, professional experience and leisure activities.

We believe that the research and knowledge of this phenomenon supports the development of health promotion strategies in the workplace, searching for a better quality of professional life and provision of quality care. Recently, different authors have emphasized the negative consequences of caring for others without taking care of oneself, pointing out the need to better articulate worker and its task in the promotion of their occupational health, as it has been advocated in nursing work. Moreover, prevention strategies for job stress among nurses can be focus on: education/training about stress and fatigue symptoms, regular monitoring (e.g. through brief questionnaires under the responsibility of occupational health services), peer-to-peer discussions and peer support (e.g. discussions of real cases with sharing of experiences, taking special care to avoid moments of personal vulnerability that may lead to professional accusations). These can be important contributions to prevent burnout and compassion fatigue, increasing professional satisfaction with patients’ care provided.

## References

[1] Elliott C (2017). Emotional labour: learning from the past, understanding the
present. British Journal of Nursing. Mark Allen Group.

[2] Ueno LGS, Bobroff MCC, Martins JT, Bueno RCRM, Linares PG, SG (2017). Occupational Stress: Stressors Referred By the Nursing
Team. J Nurs UFPE on line.

[3] Giménez-Espert MC, Prado-Gascó VJ, Valero-Moreno S (2019). Impact of work aspects on communication, emotional intelligence
and empathy in nursing. Rev. Latino-Am. Enfermagem.

[4] Adimando A (2017). Preventing and Alleviating Compassion Fatigue Through Self-Care:
An Educational Workshop for Nurses. J Holist Nurs.

[5] Wentzel D, Brysiewicz P (2017). Integrative Review of Facility Interventions to Manage Compassion
Fatigue in Oncology Nurses. Oncol Nurs Forum.

[6] Pehlivan T (2018). Compassion Fatigue: The Known, Unknown. J Psychiatric Nurs.

[7] Missouridou E (2017). Secondary posttraumatic stress and nurses’ emotional responses to
Patient’s trauma. J Trauma Nurs.

[8] Figley CR (2002). Compassion fatigue: psychotherapists’ chronic lack of self
care. J Clin Psychol.

[9] Stamm BH (2010). The Concise ProQOL Manual.

[10] Duarte J, Pinto-Gouveia J (2017). The role of psychological factors in oncology nurses’ burnout and
compassion fatigue symptoms. Eur J Oncol Nurs.

[11] Van Mol MMC, Kompanje EJO, Benoit DD, Bakker J, Nijkamp MD (2015). The prevalence of compassion fatigue and burnout among healthcare
professionals in intensive care units: A systematic review. PLoS One.

[12] Drury V, Craigie M, Francis K, Aoun S, Hegney DG (2014). Compassion satisfaction, compassion fatigue, anxiety, depression
and stress in registered nurses in Australia: Phase 2
results. J Nurs Manage.

[13] Henson JS (2017). When Compassion Is Lost. Medsurg Nurs.

[14] Sinclair S, Raffin-Bouchal S, Venturato L, Mijovic-Kondejewski J, Smith-MacDonald L (2017). Compassion fatigue: A meta-narrative review of the healthcare
literature. Int J Nurs Studies.

[15] Jarrad R, Hammad S, Shawashi T, Mahmoud N (2018). Compassion fatigue and substance use among nurses. Annals Gen Psychiatry.

[16] Ames M, Salmond E, Holly C, Kamienski M (2017). Strategies that reduce compassion fatigue and increase compassion
satisfaction in nurses. JBI Database Syst Rev Implement Reports.

[17] Guirardello EB (2017). Impacto do ambiente de cuidados críticos no burnout , percepção
da qualidade do cuidado e atitude de segurança da equipe de
enfermagem. Rev. Latino-Am. Enfermagem.

[18] Gómez-Urquiza JL, De la Fuente-Solana EI, Albendín-García L, Vargas-Pecino C, Ortega-Campos EM, Cañadas-De la Fuente GA (2017). Prevalence of Burnout Syndrome in Emergency Nurses: A
Meta-Analysis. Critical Care Nurse.

[19] Puerto J, Soler L, Montesinos M, Marcos A, Chorda V (2017). A new contribution to the classification of stressors affecting
nursing professionals. Rev. Latino-Am. Enfermagem.

[20] Hinderer KA, VonRueden KT, Friedmann E, McQuillan KA, Gilmore R, Kramer B (2014). Burnout, compassion fatigue, compassion satisfaction, and
secondary traumatic stress in trauma nurses. J Trauma Nurs.

[21] Carvalho P, Sá L (2011). Qualidade de vida profissional nos cuidados paliativos: Adaptação
Cultural e estudo de validade da escala “Professional Quality of Life 5
(ProQOL5). Inst Ciências da Saúde da Univ Católica Port.

[22] Duarte J (2017). Professional quality of life in nurses: Contribution for the
validation of the Portuguese version of the Professional Quality of Life
Scale-5 (ProQOL-5). Anal Psicol.

[23] Hunsaker S, Chen HC, Maughan D, Heaston S (2015). Factors That Influence the Development of Compassion Fatigue,
Burnout, and Compassion Satisfaction in Emergency Department
Nurses. J Nurs Scholarship.

[24] Hooper C, Craig J, Janvrin DR, Wetsel MA, Reimels E (2010). Compassion Satisfaction, Burnout, and Compassion Fatigue Among
Emergency Nurses Compared With Nurses in Other Selected Inpatient
Specialties. J Emergency Nurs.

[25] Giorgi F, Mattei A, Notarnicola I, Petrucci C, Lancia L (2018). Can sleep quality and burnout affect the job performance of
shift-work nurses? A hospital cross-sectional study. J Adv Nurs.

[26] Chen S-C, Chen C-F (2018). Antecedents and consequences of nurses’ burnout. Manage Decision.

[27] Yu H, Jiang A, Shen J (2016). Prevalence and predictors of compassion fatigue, burnout and
compassion satisfaction among oncology nurses: A cross-sectional
survey. Int J Nurs Studies.

[28] Sacco TL, Ciurzynski SM, Harvey ME, Ingersoll GL (2015). Compassion Satisfaction and Compassion Fatigue Among Critical
Care Nurses. Crit Care Nurse.

[29] Mooney C, Fetter K, Gross BW, Rinehart C, Lynch C, Rogers FB (2017). A Preliminary Analysis of Compassion Satisfaction and Compassion
Fatigue with Considerations for Nursing Unit Specialization and Demographic
Factors. J Trauma Nurs.

